# Disruption of CDK5 regulatory subunit 1, p35, limits immunosuppressive M2 macrophages while maintaining functional M1 macrophages

**DOI:** 10.3389/fimmu.2025.1584791

**Published:** 2025-09-17

**Authors:** Juliana R. Zampieri, Sung Hee Choi, Jay T. Myers, Suzanne L. Tomchuck, Saada Eid, David Askew, Alex Y. Huang

**Affiliations:** ^1^ Faculdade Israelita de Ciências da Saúde Albert Einstein Hospital Israelita Albert Einstein, São Paulo, Brazil; ^2^ Department of Pediatrics, Case Western Reserve University School of Medicine, Cleveland, OH, United States; ^3^ Case Comprehensive Cancer Center, Case Western Reserve University School of Medicine, Cleveland, OH, United States; ^4^ Center for Pediatric Immunotherapy, Angie Fowler Adolescents and Young Adults (AYA) Cancer Institute, University Hospitals (UH) Rainbow Babies & Children’s Hospital, Cleveland, OH, United States

**Keywords:** macrophage, polarization, CDK5, antigen processing, p35

## Abstract

**Introduction:**

Macrophage polarization into M1 or M2 phenotypes is a complex process influenced by various factors. However, existing literature and ongoing research support the view that Cyclin-Dependent Kinase 5 (CDK5) may play an important role in this process. CDK5 is a protein kinase that requires association with regulatory, co-activating proteins, p35 (CDK5R1) or p39 (CDK5R2), for functional activation.

**Purpose:**

This study investigated the role of the p35 protein in regulating M1 and M2 polarization.

**Methods:**

We compared bone marrow derived macrophages from wild type (WT) and p35 knockout (KO) mice under both M1 (IFNγ + LPS) and M2 (IL4) conditions, differentiated with M-CSF or GM-CSF. The expression of surface markers (CD86, CD206), enzyme expression (Arginase-1 and iNOS), metabolism and antigen process and presentation were compared.

**Results:**

While p35 had modest effect on phenotype during M1 or M2 polarization, p35 expression was important for Arginase1 induction after M2 polarization. The absence of p35 significantly increased glycolysis during M1 polarization, while it also enhanced mitochondrial oxidative phosphorylation in the context of M2 polarization. While p35 was important for antigen processing by M0 and M2, M1 were able to maintain capacity to process antigen albeit with a reduction due to decreased stability of peptide: MHC II complex.

**Conclusion:**

While loss of p35 resulted in minor changes in phenotype, there were decreases in ARG-1 production and STAT3 phosphorylation, increased metabolism, and dramatically reduced antigen processing by M0, M1 or M2. The absence of p35 enhanced antigen uptake, but it had no effect on degradation of antigen, suggesting an inability to produce peptide: MHC II complexes in the absence of p35 in M0 and M2. In contrast, p35-deficient M1 maintained an ability to rapidly produce peptide: MHC II complexes but showed a reduction in the stability of these complexes on the surface. Our findings reveal a crucial role for p35 in regulating macrophage metabolism and antigen function, with implications for the development of novel therapeutic strategies.

## Introduction

The ability of macrophages (Mϕ) to respond to their environment by undergoing phenotypic and functional transformation is essential in regulating host response to injuries, infections, or malignancies with the ultimate goal of clearing pathogenic challenges and restoring tissue homeostasis. Through the use of multiple surface receptors, Mϕ detect the presence of damaged tissues or cellular debris through receptors for danger associated molecular patterns, or bacterial or viral components through pathogen associated molecular patterns (PAMPs) (1, 2). These receptors include the family of Toll-like receptors, (TLRs), NOD-like receptors, C-type lectin receptors, and retinoic acid-inducible gene like receptors (3). Activation of Mϕ is further enhanced by the production of IFNγ , by other innate immune cells including natural killer cells in response to the same pathogens (4). Traditionally, Mϕ that have been exposed to a combination of PAMPs and IFNγ are classified as an activated, inflammatory M1 phenotype. These M1 Mϕ express high levels of the costimulatory molecules CD80 and CD86, with enhanced expression of MHC II, and produce various soluble factors including inducible nitric oxide synthase (iNOS), TNFα, IL1β, IL6, and IL12 (5). The upregulation of CD274 (PDL1) is also characteristic of inflammatory Mϕ (6). Alternatively, Mϕ can also be activated by other factors resulting in anti-inflammatory M2 phenotype. Mϕ that have been cultured in the presence of IL4 or IL13 are referred to as M2 Mϕ and are characterized by high expression of CD206, decoy receptor IL1R, IL1R antagonists, as well as STAT6, GATA3, SOCS1, CD163, CD36, and Arginase 1 (Arg1). While STAT1 has been associated with M1 polarization, in addition to STAT6, STAT3 also plays a role in M2 polarization (7). Both the IL10/STAT3 (8) and IL6/STAT3 (9) pathways promote the M2 phenotype. While STAT3 is mainly associated with anti-inflammatory responses, it also can contributes to M1 polarization through the regulation of pro-inflammatory cytokines (10).

Besides differing profiles in surface molecular phenotypes and cytokine production, M1 and M2 Mϕ also differ in their basic metabolism. M1 Mϕ preferentially utilize the glycolytic and pentose phosphate pathways for enhanced production of NADPH essential to produce reactive oxygen species (ROS) and nitric oxide (NO) (11). The TCA cycle is interrupted by two reactions mediated by isocitrate dehydrogenase and succinate dehydrogenase, resulting in the accumulation of citrate and succinate (12), in M1 Mϕ, leading to production of itaconic acid, a metabolite with anti-microbial activity and participates in both fatty acid biosynthesis and NO production. On the other hand, M2 Mϕ rely predominantly on fatty acid oxidation (FAO) with intact TCA cycle that utilizes the electron transport chain (ETC) with a high rates of mitochondrial oxidative phosphorylation (OXPHOS). Further, glutamine catabolism is essential for M2 Mϕ polarization (13). As M1 Mϕ are often associated with a strong anti-tumor function, it is generally thought that the tumor microenvironment (TME) promotes the establishment of M2-like Mϕ that are functionally anti-inflammatory, thereby supporting tumor growth and suppressing anti-tumor immune responses.

M-CSF and GM-CSF play essential roles for both maintaining steady-state Mϕ and directing responses to immune challenge. Bone marrow-derived macrophages (BMDMs) exposed to GM-CSF (GM-BM) tend to show greater propensity to produce inflammatory cytokines such as TNFα, IL6, or IL12p70, while BMDMs grown in M-CSF (M-BM) tend to produce IL10 or CCL2 (14). M-CSF, but not GM-CSF, is detected in most tissues under steady state conditions; the production of GM-CSF requires an inflammatory stimulation (15). Previous proteomic analyses showed that GM-BM had enriched glycolytic capacity while M-BM showed increased endocytosis, suggesting that M-BM are primed more toward homeostatic function (16). Furthermore, M-CSF may also support the polarization and survival of tumor-associated Mϕ (TAM) (17).

Mϕ are well known as antigen presenting cells and M1 Mϕ have enhanced antigen-presenting function (18). Antigen processing is defined as a process that results in the generation of peptide:MHC II molecular complexes on the surface of the antigen presenting cell (APC). The antigens are generated from both particulate and soluble forms and can derive from either endogenous or exogenous cellular sources. In the case of classical exogenous antigen processing and presentation on MHC II molecules, the antigens are endocytosed through non-specific mechanisms such as micropinocytosis, phagocytosis, autophagy or via receptor-mediated processes where they are incorporated into intracellular vesicles (19). These vesicles are transported within the cytoplasm and fused with other internal vesicles that follow the early endosomal – late endosomal – lysosomal trafficking axis. These vesicles contain components of antigen processing machinery including proteases and nascent MHC molecules that undergo increasing acidification resulting in enhanced denaturation and proteolytic activity. MHC II antigen processing can utilize both newly synthesized MHC II as well as recycled molecule, resulting in the expression of peptide:MHC II complexes on surface of APCs.

Cyclin-dependent kinase 5 (CDK5) is a proline-directed serine-threonine kinase that requires association with one of two major regulatory co-activators, p35 (CDK5R1) or p39 (CDK5R2), to be activated (20). CDK5 was first described in the developing brain where the loss of CDK5 was associated with embryonic lethality due to the formation of abnormal structures in cerebral cortex with inverted neurogenic gradient (21). Within the hematopoietic system, CDK5 was reported to play an important role in T cell migration, proliferation, and cytokine production in both autoimmune disease and graft-versus-host disease models (22, 23). While p39 can partially substitute for loss of p35 in neurons, loss of p35 shows similar defects as those observed in CDK5 knockout (KO) mice in the development of pathogenic T cells, suggesting that p39 is unable to replace p35 in lymphocytes development and function. CDK5 is also associated with the release of secretory vesicles from neutrophils (24) and contributes to neutrophil survival during inflammation through its action on myeloid cell leukemia-1 (MCL-1) (25). Treatment of BMDM with roscovitine (a non-specific inhibitor of CDK2, CDK5, and CDK9) reduced the production of NO (26), and the deletion of CDK5 enhanced the anti-inflammatory effect of dexamethasone (27). In Mϕ differentiated with GM-CSF, p35 increased in response to LPS stimulation while the loss of p35 resulted in delayed production of IL10 (28). Recently, it was suggested that CDK5 regulates the early production of IL10 in M1 Mϕ through c-MAF (c-Musculoaponeurotic fibrosarcoma) (29). CDK5 has multiple targets in many tissues including STAT3 (30) and PPARγ (31), how CDK5 modulates Mϕ effector function has yet to be fully explored.

In this study, we examined the role of CDK5RP1 (p35) in regulating M1 and M2 Mϕ by evaluating phenotypic changes, cytokine production, and arginine utilization either through iNOS or ARG-1 under M1 or M2 polarization conditions. Since deletion of CDK5 results in embryonic lethality, we utilized genetic deletion of p35 as a model of CDK5/p35 disruption without using CDK5 inhibitors which are known to also disrupt other CDKs critical for cellular function. We found that loss of p35 resulted in only minor changes in phenotype and cytokine production, decreased ARG-1 production and STAT3 phosphorylation, increased glycolysis and mitochondrial OXPHOS, and reduced effective antigen processing by M0, M1 and M2 Mϕ subtypes. Loss of p35 enhanced antigen uptake without an effect on degradation of antigen, suggesting an inability to produce peptide:MHC II complexes in the absence of p35 in M0 and M2 Mϕ. In contrast, p35-deficient M1 Mϕ retained the ability to rapidly produce peptide:MHC II complexes but showed a reduction in the stability of these peptide:MHC II complexes on the Mϕ surface. Understanding the impact of CDK5/p35 on Mϕ polarization could have therapeutic implications, including anti-tumor responses, when it could maintain M1 but downregulate the M2 population.

## Methods

### Mice

p35 wild-type (WT) or p35 knockout (KO) mice were generated by breeding heterozygous p35^+/-^ mice, initially provided by Dr. T. Pareek (23). Animals were housed, bred, and handled in the Animal Resource Center facilities at Case Western Reserve University according to approved Institutional Animal Care and Use Committee protocols (# 2015-0118) and in accordance with American Association for Accreditation of Laboratory Animal Care and NIH guidelines.

### Culture and polarization of bone marrow-derived Mϕ

Bone marrow cells were collected from tibia and femur of WT or KO mice as previously described (32), and the cell suspension was filtered through a 70 μm cell strainer (VWR, Radnur, PA) into a 50 mL tube and the cell pellet was collected by centrifugation at 1600 RPM for 5 minutes. Red blood cells were lysed by incubating for 5 minutes with ACK lysis buffer (Life Technologies, Grand Island, NY), and then the cells were washed and seeded at (0.5–1 x 10^6^ cells/mL) in a 100 mm tissue culture dish containing DMEM media containing L-glutamine (Life Technologies) supplemented with 100 U/mL penicillin/streptomycin, 10% fetal bovine serum (FBS; GIBCO Invitrogen), NEAA, HEPES buffer, and Sodium Pyruvate (Life Technologies). Complete DMEM was then supplemented with either 25-30% LADMAC supernatant for M-CSF (33) or murine GM-CSF (25 ng/mL; Miltenyi Biotec, Gladbach, Germany). The cell culture was replenished with fresh supplemented media every 3–4 days. After 7–10 days, cells were transferred to 6-well plates (1x10^6^ cells/mL), incubated for an additional day with LADMAC or GM-CSF containing media, and then switched to complete DMEM without growth factors before activation. At this stage we have generated either M-CSF-derived bone marrow Mϕ (M-BM) or GM-CSF-derived BM Mϕ (GM-BM). Mϕ were then polarized with IFNγ (100 ng/mL, Biolegend, San Diego CA) and/or Ultrapure LPS (100 ng/mL, InvivoGen, San Diego CA) for M1 conditions, or with IL4 (25 ng/mL, Biolegend) for M2 conditions.

### Flow cytometry

BMDM cells were added at 1–3 x10^5^ in V-bottom 96-well plates in FACS buffer (0.1% BSA, 2.5 mM EDTA in PBS, 1:100 anti-mouse CD16/32) for 20 minutes at 4°C. Cells were washed and incubated with anti-CD86-FITC, anti-F4/80-AlexaFluor 700, anti-CD206-PE, anti-CD274-PECy7, Zombie NIR (Biolegend) and anti-MHC II (I-A/I-E)-PE Fluor 610 (eBioscience, San Diego CA) for 30 minutes. For intracellular staining 2 x 10^5^ cells were fixed in Cyto-Fix/Perm buffer (Biolegend) for 20 minutes at room temp, and washed with Perm Wash buffer prior to addition of anti-Arginase1-PE (Biolegend) for 20 minutes. Antibody concentrations were determined by titration and samples were collected using a CytoFLEX flow cytometer (Beckman Coulter, Indianapolis, IN) and analyzed using FlowJo (FlowJo, Ashland OR). Unstained and isotype controls were used to determine gating strategy while single color stained UltraComp eBeads (Thermo Scientific) were used for multicolor compensation.

### ELISA

Mϕ were seeded in 24-well plates at 5 x10^5^ cells per 500 μl in medium and stimulated under M1 or M2 polarizing conditions for 24 hours. Cell culture medium was then collected, and the levels of IL10, and IL1β levels in the BMDM culture supernatant were measured by ELISA (IL1β: Cat# DY401, and IL10: Cat# DY417 R&D, R&D Systems, Minneapolis MN) according to manufacturer’s instructions.

### Western blotting

Mϕ were lysed in RIPA lysis buffer (Sigma-Aldrich, St. Louis MO R0278) supplemented with Halt Protease Inhibitor Cocktail 100x (Thermo Fisher, 78430). Lysates were placed on ice for 15 minutes then centrifuged at 13,000 RPM for 15 minutes at 4°C. Protein concentrations of lysates were then determined using the DC Protein Assay (Bio-Rad Hercules, CA, 5000111). Samples were heated to 95°C for 10 minutes in Laemmli sample buffer under reducing conditions and then loaded on 4-12% Bis-Tris gels and electrophoresed at 100 V for 40–50 minutes. Proteins were then transferred to nitrocellulose membranes and probed using the following rabbit antibodies from Cell Signaling Technology (CST, Danvers, MA) ARGINASE-1 (#93668S), iNOS (#13120S), Phospho-STAT3 (Ser727 - #9134S), Phospho-STAT3 (Tyr705 - #9145S), STAT3 (#4904S), CDK5 (#14145S), p35/25 (#2680S), β-ACTIN (#4970S) and goat-anti-rabbit HRP Conjugate (#5125S). Antigen detection was performed using ProSignal^®^ Pico ECL Reagent Cat #: 20-300 (Genesee Scientific, San Diego, CA). ImageJ software was used to quantify band intensities and normalize protein expression levels relative to β-actin. In each Western blot, protein levels in the wild-type (WT) group were assigned a reference value of 1, and the relative expression in the p35 knockout (KO) group was calculated based on this reference.

### qPCR

Total RNA from WT and p35 KO BMDM was isolated using the Cytiva illustra™ RNAspin Mini Isolation Kit (Fisher Scientific, CAT 25050071) and cDNA was produced with the EasyScript cDNA Synthesis Kit (Lambda Biotech, Miami, FL, CAT#G234). Real-time RT-PCR analysis was performed using the TaqMan assay (Applied Biosystems, Waltham, MA), *Arg1* TaqMAN primer/probe (Mm00475988_m1), and *iNos2* TaqMAN primer/probe (Mm00440502_m1). Relative mRNA expressions were analyzed using the ΔΔCT method and normalized to expression of the *Gapdh* housekeeping gene (Applied Biosystems), fold change in gene expression was determined using WT M0 as the control.

### Seahorse assay

The Cell Mito Stress Test was performed according to the Agilent Technologies protocol. Briefly, BMDMs were plated in a 96-well Seahorse assay plate at a density of 10_5_ cells per well in Seahorse Assay Media. Cells were either left untreated or stimulated under M1 or M2 conditions for 24 hours at 37 °C. The following day, the plate was incubated for 1 hour at 37 °C in a non-CO2 incubator. For the Cell Mito Stress Test, mitochondrial modulators were sequentially injected as follows: 1 μM oligomycin (Sigma-Aldrich O4876-5MG), 3 μM FCCP (Sigma-Aldrich C2920-10MG), 100 nM antimycin A (Sigma-Aldrich A8674-25MG), and 1 μM rotenone (Sigma-Aldrich R8875). The Seahorse XFe96 Extracellular Flux Analyzer (Agilent Technologies, Santa Clara, CA) continuously measured the rate of oxygen consumption (OCR) and the rate of proton production or extracellular acidification rate (ECAR), which directly quantify oxidative phosphorylation (OXPHOS) and glycolysis, respectively.

### Immunofluorescent bead uptake

BMDM from p35 WT or p35 KO mice were cultured under M0, M1, or M2 polarizing conditions for 24 hours and 5 x 10^5^ Mϕ were placed at 37°C for 30 minutes. Ovalbumin (OVA)-coated immunofluorescent beads (Fluoresbrite Carboxylate – Polysciences Warrington PA Cat# 17797-1) (34) were added to M0, M1, or M2 Mϕ cultures at an equivalent dose of 100 µg/ml OVA antigen. Samples were collected every 15 minutes, placed on ice, and then washed and examined by flow cytometry.

### Degradation of ovalbumin

To measure degradation of OVA, 10 μl of 1 mg/mL DQ-OVA (Invitrogen, Waltham, MA) was added to 1 x 10^6^ Mϕ that were pre-warmed to 37°C for 15 minutes. Cells were washed twice in ice-cold complete DMEM, resuspended in warm complete DMEM, samples were removed either immediately or every 15 minutes, and transferred to 96-well V-bottom plate containing ice cold PBS. Fluorescence was measured by flow cytometry.

### Antigen processing assay

To measure antigen processing, Mϕ were cultured under polarizing conditions for 24 hours then washed. 1 x 10^5^ cells were added per well of a 96-well plate. Different concentrations of soluble ovalbumin (Wothington #3054, Lakeland NJ) or ovalbumin-coated latex beads (L-OVA, Polybead microsphere Polysciences Cat#19814-15) (32) were added to Mϕ cultures and cultured with 1 x 10^5^ DOBW cells, a T cell hybridoma that recognizes OVA_323–339_ presented via I-A^b^ (35). Supernatant was collected 24 hours later and stored at -20°C. IL2 levels in supernatant were measured using ELISA (Biolegend Cat#431001) according to manufacturer’s protocols. For measuring kinetics of antigen processing, 1 x 10^5^ polarized Mϕ were added per well of a 96-well plate. L-OVA were added to Mϕ cultures at 100 μg/mL for 1 hour at 37°C. At 20-minute intervals thereafter, cells were washed with DMEM and fixed with 0.5% paraformaldehyde (PFA) in DMEM at room temperature for 20 minutes. Following fixation, cells were washed 3 times in DMEM, and then incubated with 0.2 M D, L-lysine for an additional 20 minutes. These wells were then washed 3 times with DMEM. Cells were then cultured with 1 x 10^5^ DOBW cells overnight and the supernatant was collected 24 hours later.

### Peptide: MHC II complex stability assay

To determine stability of peptide:MHC II complexes, Mϕ were cultured under polarizing conditions for 24 hours then washed. 1 x 10^5^ cells were added per well of a 96-well flat bottom plate and cells were incubated with 3 μM OVA_323–339_ peptide for varying durations. Cells were washed with 10% FBS-DMEM and fixed at 20-minute intervals with 0.5% PFA in DMEM for 20 minutes at room temperature. Following fixation, cells were washed 3 times in DMEM, and then incubated with 0.2 M D, L-lysine for an additional 20 minutes. These wells were then washed 3 times with DMEM. Cells were then cultured with 1 x 10^5^ DOBW cells overnight and the supernatant was collected 24 hours later. Half-life was determined using the formula t_1/2_ = t/log_1/2_(N(t)/N_0_).

### Statistical analysis

Data shown were the means ± SEM or SD, as indicated. Data between two groups were analyzed by unpaired *t*-test (Prism 6.0, GraphPad, Boston, MA). Comparisons among three or more groups were performed using one-way ANOVA, followed by Tukey’s *post hoc* test for multiple comparisons, which allows the identification of specific group differences.

## Results

### Differential impact of p35 in LPS and LPS+IFNγ polarized M1 Mϕ differentiation

**Figure 1:**
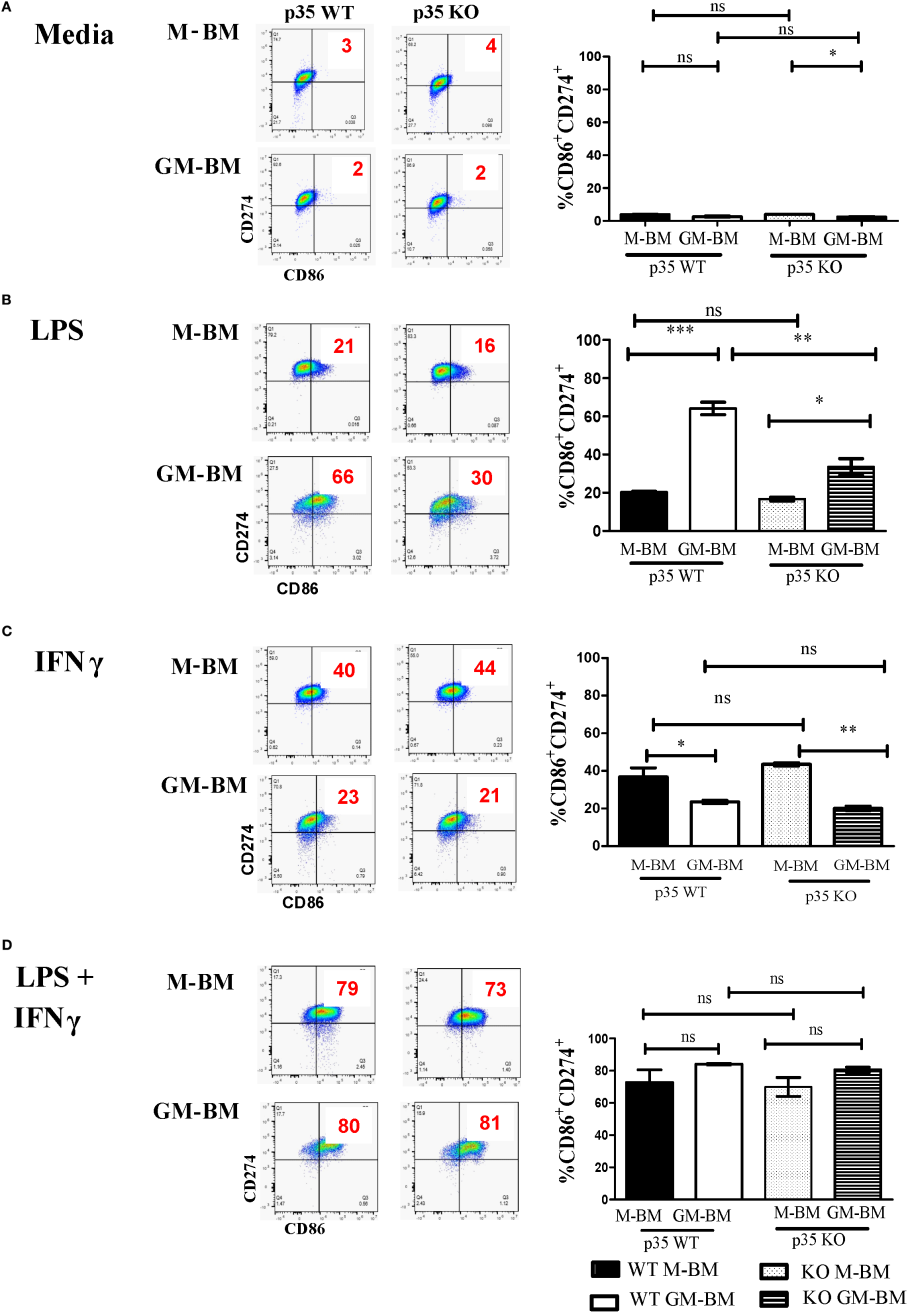
p35 is important for LPS-mediated M1 polarization of GM-BM. **(A)** M-BM (BMDM grown from M-CSF) or GM-BM (BMDM from GM-CSF) from p35 WT or KO BM were cultured in **(A)** Media only, **(B)** LPS, **(C)** IFNγ, or **(D)** IFNγ + LPS for 24 hours before being subjected to flow cytometry analysis. Two experiments were performed and percentage of CD86^+^CD274^+^ cells were determined. One-Way ANOVA, Turkey’s multiple comparison: *p <.05; **p <.01; ***p <.001; ns, not significant.

To more precisely investigate the contribution of functional CDK5/p35 complex in Mϕ polarization under homeostatic and inflammatory conditions, we used BMDM from wildtype (p35 WT) or p35^-/-^ (p35 KO) mice cultured in the presence either M-CSF or GM-CSF, then analyzed the effects of p35 on M1 polarization in the presence of LPS, IFNγ or both. While most BMDM cultured with M-CSF (M-BM) or GM-CSF (GM-BM) alone were CD274^+^, few cells expressed CD86 at baseline (**Figure 1**). Thus, examining expression of CD86^+^CD274^+^ Mϕ was a better indicator of the presence of M1 Mϕ. LPS alone significantly induced higher CD86^+^CD274^+^ expression in GM-BM than M-BM from p35 WT mice; however, this induction of CD86^+^CD274^+^ in GM-BM was significantly suppressed in cells from p35 KO mice, while the same populations were relatively unaffected from p35 WT and KO donors when they were cultured under M-BM conditions (**Figure 1**). Culturing with IFNγ alone resulted in similar higher expression of CD86 among M-BMs from both mouse strains as compared to GM-BM (**Figure 1**), with p35 not playing a significant role in these differential expressions. The addition of both IFNγ + LPS resulted in a dramatic upregulation of CD86^+^CD274^+^ in both M-BM and GM-BM to a similar degree (**Figure 1**) irrespective of functional p35. We also evaluated effects of LPS and INFγ concentrations on the induction of CD86 expression, with both 10 ng and 100 ng of INFγ inducing maximal CD85 expression levels (**Supplementary Figure 1**). These results suggest that LPS-induced M1 polarization specifically under GM-CSF culture conditions is uniquely dependent on the presence of p35, whereas signals induced by IFNγ or IFNγ + LPS can overcome the dependency of CD86 upregulation on a functioning CDK5/p35 complex, even though TLR4 expressions could not be readily detected by flow cytometry in WT and KO M0 Mϕ, while these cells constitutively expressed CD14 that could be enhanced under M1 conditions (**Supplementary Figure 2**).

Next, we investigated IL4-induced M2 polarization by examining CD206 expression among BMDMs (**Supplementary Figures 3A, B**). IL4 dramatically and equally increased CD206 expression in M-BM from both p35 WT and p35 KO mice, demonstrating that loss of p35 had no significant effect on CD206 expression. Similarly, culturing of Mϕ in GM-CSF (GM-BM) had no effect on CD206 expression under IL4-induced M2 conditions (*data not shown*). With only modest differences seen between M-BM and GM-BM, we focused subsequent analyses only on M-BM population as it better represented Mϕ under physiologic homeostatic conditions.

### p35 KO reduced ARG-1 production and STAT3 phosphorylation under M2 conditions

**Figure 2:**
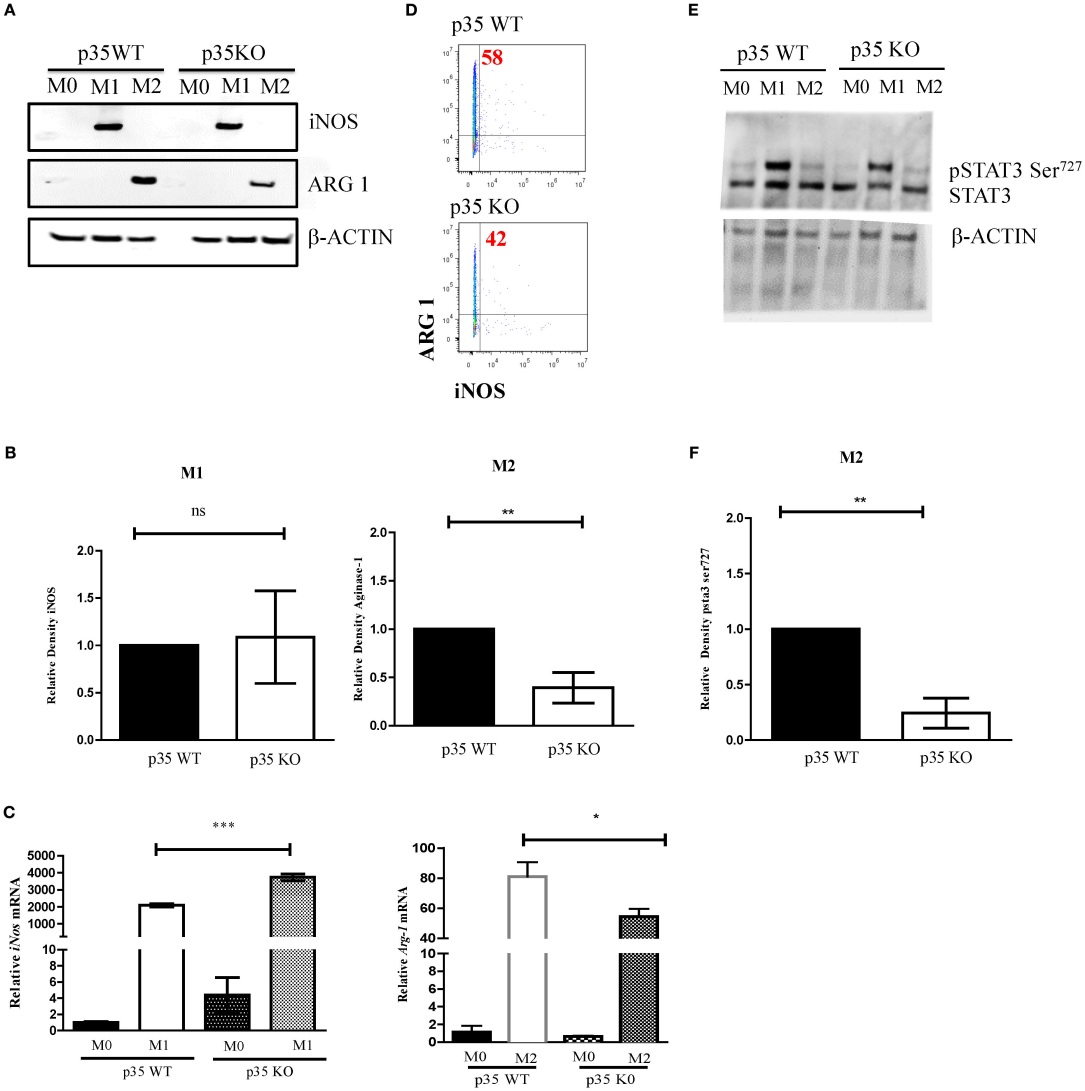
Absence of p35 reduces ARG-1 production and STAT3 phosphorylation in M2 Mϕ. BMDM from WT and p35 K0 were cultured under M0, M1, or M2 conditions for 24 hours then the protein lysates were analyzed via Western Blot for ARG-1, iNOS, p-STAT3, or β-ACTIN **(A, E)**. Image J was used to compare the band density and normalize to the amount of β-actin **(B, F)**. Cells were also analyzed by qPCR to determine the relative abundance of *iNos* and *Arg-1* normalized to that of *Gapdh* mRNA **(C)**. Intracellular staining of M2 polarized p35 WT and KO Mϕ stained with anti-Arginase1-PE and analyzed by flow cytometry **(D)**. Comparison between p35 WT and p35 KO were performed using unpaired T test. *p <.05; **p <.01; ***p <.001; ns, not significant. A total of 3 to 4 experiments were performed for qPCR or Western blot analysis.

In addition to phenotypic analysis, we evaluated the impact of p35 during M1 and M2 polarization on the production of iNOS and ARG-1, as both proteins are important in the utilization of arginine but have opposing effects in the tissue environment in response to polarization. We found no significant differences in the production of iNOS under M1 polarizing conditions between WT and p35 KO Mϕ (**Figure 2**), even though there was significant increase in iNOS mRNA expression in p35 KO Mϕ. However, ARG-1 was significantly lower (p<0.01) in the p35 KO when compared to p35 WT under M2 polarization. This was true in both protein (**Figure 2**; p>0.05) and gene expression (**Figure 2**; p<0.01). We were able to further validate this decrease in ARG-1 expression in p35 KO Mϕ by flow cytometry (**Figure 2**). These data further support our hypothesis that, in the absence of p35, Mϕ remain capable of polarizing functionally towards M1 phenotype but exhibit a diminished M2 functional polarizing capability.

STAT3 plays a critical role in both M1 and M2 polarization (36), and it has been shown to be regulated by CDK5 via phosphorylation of serine 727 (30, 37). In p35 KO, we observed that STAT3 phosphorylation was maintained under M1 polarizing conditions but was significantly decreased under M2 polarization (p<0.01) (**Figures 2E**). Altogether, the absence of p35 appears to be critical for M2 Mϕ differentiation but is dispensable for M1 Mϕ formation.

### Loss of p35 enhanced Mϕ glycolysis and OXPHOS following both M1 and M2 polarizations

**Figure 3:**
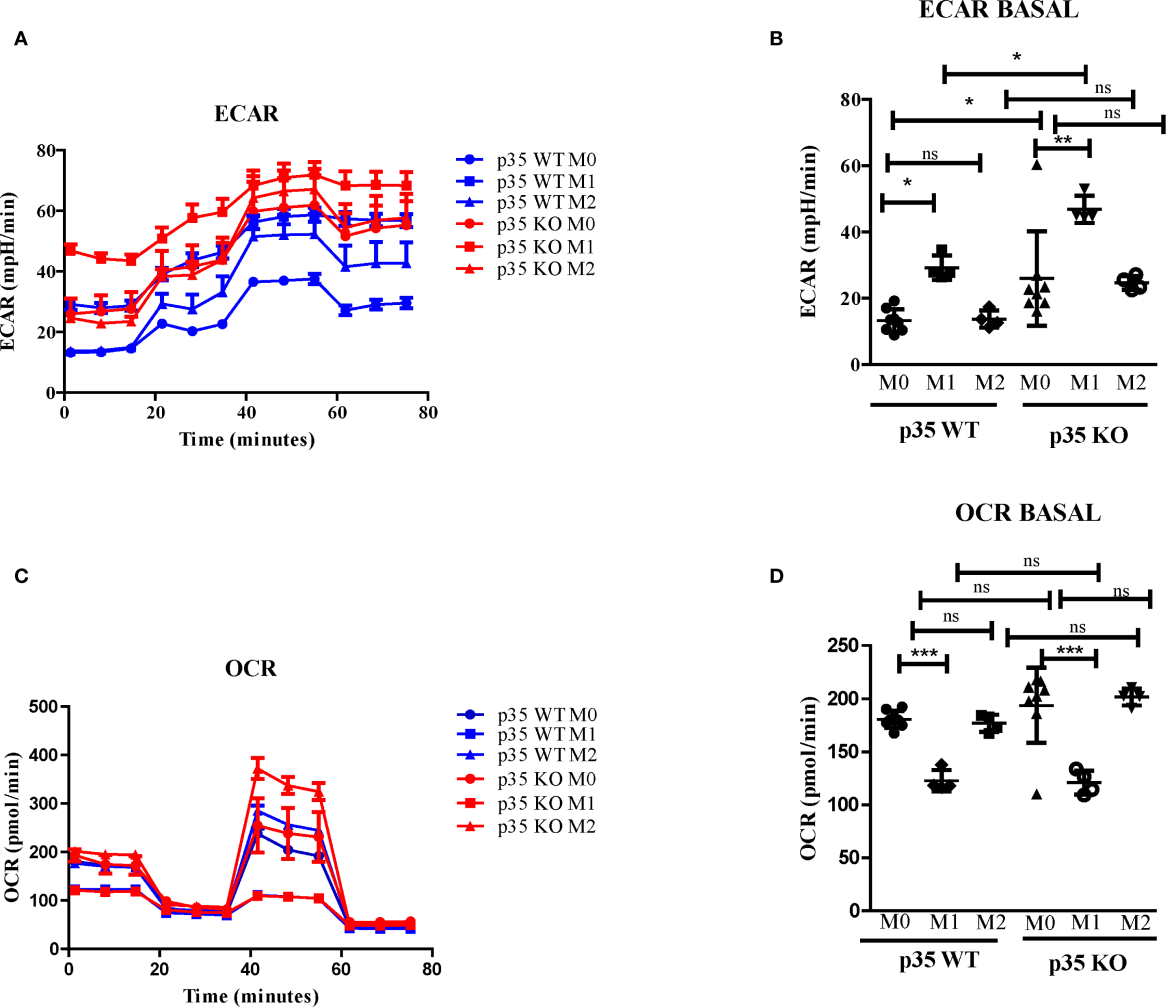
Loss of p35 expression increases OCR and ECAR expression in polarized Mϕ. BMDM were plated at 5 x 10^5^ cells per well and cultured under M0, M1, or M2 polarizing conditions. **(A, B)** Extracellular acidification rate (ECAR) and **(C, D)** oxygen consumption rate (OCR) were measured using a Seahorse analyzer were repeated 4 times with representative experiment shown. Comparison between p35 WT and p35 KO were performed One-Way ANOVA, Turkey’s multiple comparison. *p <.05; **p < .01; ***p < .001; ns, not significant.

**Figure 4:**
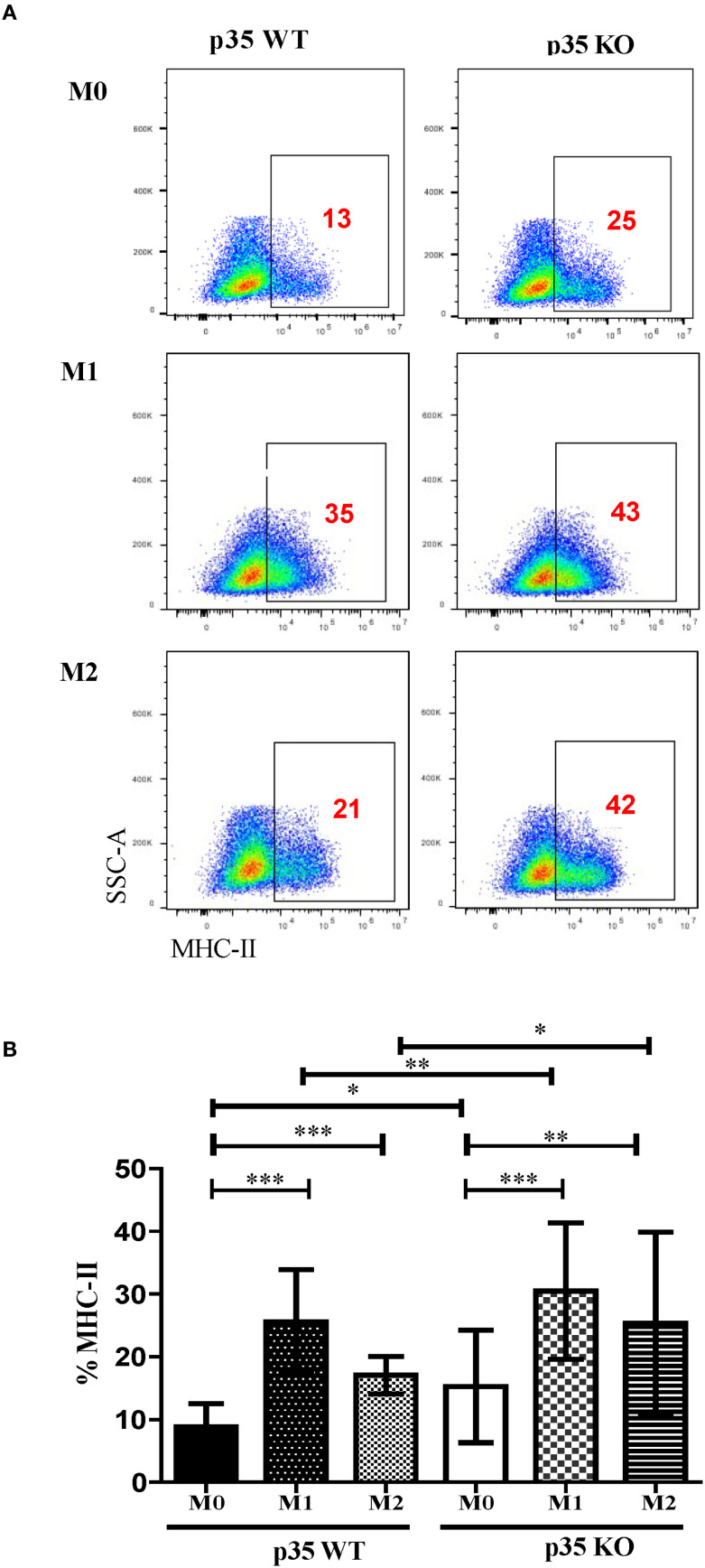
Loss of p35 expression enhances MHC II induction. **(A)** BMDM from p35 WT or p35 KO mice were cultured under M0, M1 or M2 conditions for 24 hours and examined for expression of MHC II. **(B)** Relative expression of MHC II in BMDM was determined over 2 experiments. Data between groups were analyzed using One-Way ANOVA, Turkey’s multiple comparison test. *p <.05; **p <.01; ***p <.001.

Since the phosphorylation of STAT3 at serine 727 has been associated with mitochondrial function (38), we evaluated the effects of p35 expression on M1 and M2 metabolism between p35 KO and p35 WT mice. Amino acid metabolism and the balance between glycolysis and mitochondrial OXPHOS are characteristics that help define M1 and M2 phenotypes and regulate their respective functions (39, 40). In general, M1 Mϕ have an enhanced glycolytic metabolism, while M2 Mϕ have enhanced mitochondrial OXPHOS (41). Seahorse analysis revealed that ECAR values were higher under M1 conditions relative to M0 and M2 conditions in p35 WT, and the loss of p35 expression further increased ECAR in M0 and M1 conditions (**Figures 3**, **4B**). Basal oxygen consumption rate (OCR) analysis revealed a significant reduction in M1 macrophages compared to M0 in the WT group. Similarly, in the absence of p35 M1 macrophages exhibited significantly lower OCR levels than M0 cells. No significant differences were observed among the other conditions. (**Figures 3C**). These results suggest that in the absence of p35, BMDMs are more metabolically active, exhibiting increased glycolysis under M1 polarization and enhanced OXPHOS under M2 polarization (42).

### Loss of p35 enhanced MHC II expression under both M1 and M2 polarizing conditions, but dramatically reduced antigen processing

**Figure 5:**
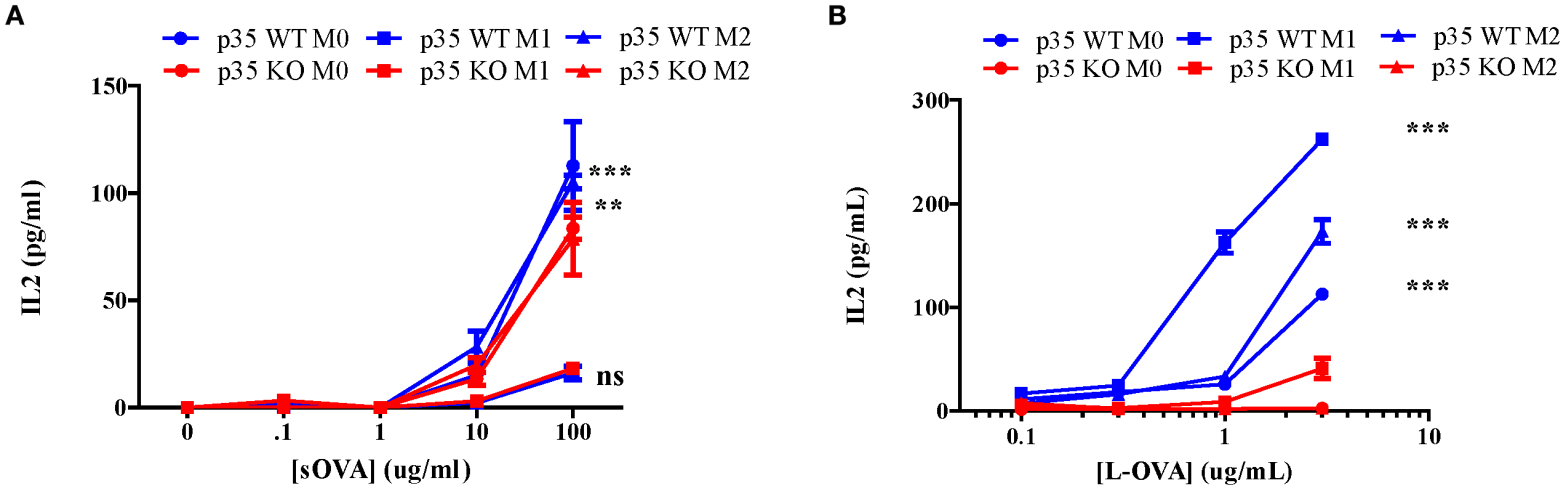
Loss of p35 reduces antigen processing. BMDM from p35 WT or KO mice were cultured for 24 hours under M0, M1, or M2 polarizing conditions and Mϕ were pulsed with different concentrations of sOVA **(A)** or L-OVA **(B)** and cultured with the T cell hybridoma DOBW for 24 hours. Supernatants were then collected and examined for IL2 production to determine relative peptide:MHC II complexes presented on BMDM. Comparison between p35 WT and p35 KO were performed using a paired T test ***p <.001, **p<.05. Shown is a representative of 4 different experiments.

Thus far, we have shown that p35 predominantly affected M2 function and phenotypic polarizations of Mϕ. To further examine the impact of p35 on the ability of Mϕ to interact and activate T cells, we observed the expression of MHC II complexes in different Mϕ populations via flow cytometry (**Figure 4**). In all experimental conditions (M0, M1, M2), Mϕ from p35 KO mice exhibited higher expression of MHC II compared to p35 WT mice. Next, we assessed the ability of these polarized Mϕ to process either soluble or particulate antigens and functionally activate T cells through the antigen processing and presentation machinery. To do so, we used varying amounts of soluble ovalbumin (sOVA) or latex beads coated with chicken ovalbumin (L-OVA) to simulate encounters with particulate antigens by Mϕ and checked for subsequent phagocytosis, antigen processing and presentation to CD4^+^ T cells over a 24-hour period. We co-cultured sOVA or L-OVA exposed Mϕ with OVA-specific, MHC II restricted CD4^+^ T cell hybridoma, DOBW for 24 hours and measured IL2 production in response to engagement by the T cell receptor (32, 34, 43, 44). While neither p35 WT nor p35 KO M1 Mϕ could process sOVA (**Figure 5**), both M0 and M2 Mϕ could with better presentation seen by p35 WT Mϕ (**Figure 5**). We also found that p35 WT Mϕ showed significantly better processing of particulate L-OVA for presentation of peptide:MHC II complexes to the T cell hybridoma over a 24-hour period than their KO counterparts, with M1 Mϕ being the best stimulator of DOBW (**Figure 5**).

### Loss of p35 enhances antigen uptake, but fails to allow for antigen processing under M0 or M2 conditions

**Figure 6:**
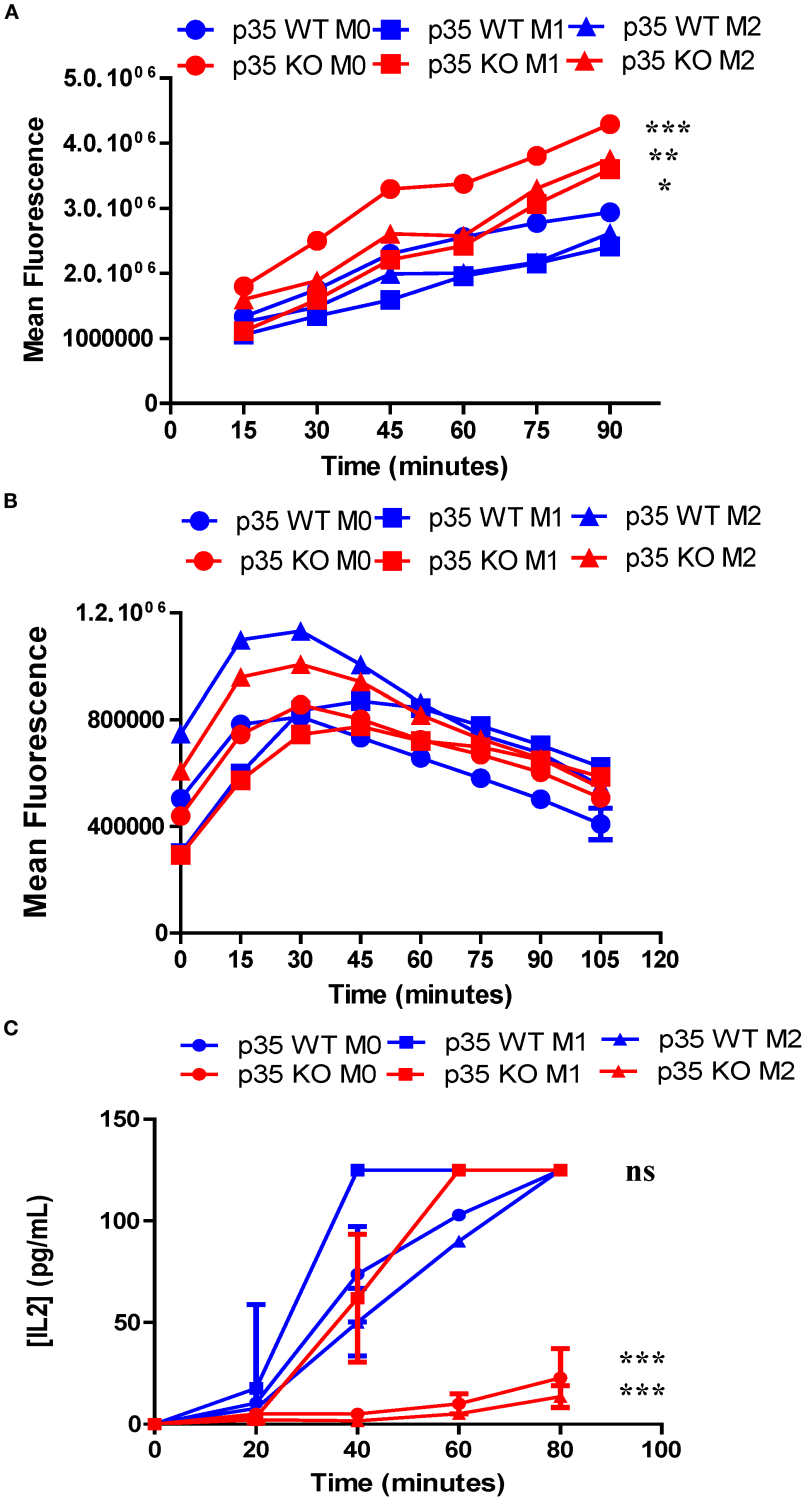
Loss of p35 enhances uptake of particulate antigen but reduces presentation of peptide:MHC II complexes under M0 and M1 conditions. BMDM from p35 WT and KO mice were cultured for 24 hours under M0, M1, or M2 polarizing conditions. **(A)** Cells were then transferred to Eppendorf tubes and placed at 37°C for 1 hour. Fluorescent beads (1 ug/ml) were then added to Mϕ, cells were examined for bead uptake in 15-minute increments using flow cytometry. **(B)** Cells were placed in Eppendorf and incubated at 37°C for 1 hour and then 10 μg of DQ-OVA was mixed with cells and incubated at 37°C for 15 minutes before being washed 2X with ice cold complete DMEM. Degradation of DQ-OVA was measured in 15-minute increments via fluorescence expression by flow cytometry. **(C)** Cells were then transferred to a 96-well plate and incubated at 37°C for 1 hour. Cells were pulsed with 100 ng/mL L-OVA for 20 minutes, washed and then fixed either immediately or in 20-minute intervals. The T cell hybridoma, DOBW, was added to wells and co-cultures were incubated at 37°C for 24 hours and supernatants were analyzed for IL2 production by ELISA. Comparison between p35 WT and p35 KO were performed using a paired T test. *p <.05; **p <.01; ***p <.001; ns, not significant. Shown is a representative of 4 different experiments.

To understand how p35 expression can influence generation of peptide:MHC II, we examined steps involved in the generation of peptide:MHC II complexes, including: 1) the ability of cells to internalize proteins, 2) processing engulfed proteins into peptide fragments, and 3) presenting these peptide:MHC II complexes on the cell surface for interaction with T cells. To assess if the reduction in antigen processing and presentation was due to decreased ability to internalize particulate antigens, we cultured M0, M1, or M2 Mϕ from p35 WT or p35 KO mice with OVA-coated fluorescent beads at 37°C *in vitro* and analyzed Mϕ for fluorescence intensity every 15 minutes by flow cytometry. We found that p35 KO M0 Mϕ showed increased bead internalization compared to p35 WT Mϕ, when compared under M0, M1, or M2 conditions, while M1 and M2 polarizing conditions resulted in a reduction of antigen internalization in both p35 WT and p35 KO Mϕ (**Figure 6A**). To determine if p35 status could influence the rate of degradation of internalized particulate proteins into peptides, M0, M1, or M2 Mϕ from p35 WT or KO mice were pulsed with DQ-OVA and the rates of degradation were followed using flow cytometry, as DQ-OVA contains a self-quenching signal that is lost after the protein undergoes degradation. While we found that initial immunofluorescence from DQ-OVA degradation may reflect CD206 expression which was influenced by M2 polarization and protein internalization rates (45), there was no difference in the rate of DQ-OVA degradation over time (**Figure 6**). Next, we examined how quickly a specific peptide:MHC II complex can be created by the differentially polarized Mϕ subsets in p35 WT and p35 KO mice. We cultured polarized Mϕ with 100 ng/mL of L-OVA for 20 minutes, after which the Mϕ were washed and incubate at 20 minute intervals for up to 80 minutes. Mϕ were then fixed with 0.5% PFA (32, 44) and co-cultured with DOBW for 24 hours and the amount of IL2 produced was determined. We found that p35 WT M1 Mϕ reached half-maximal IL2 production within 25 minutes, while M0 Mϕ and M2 Mϕ reached half-maximal IL2 production by 40 and 50 minutes, respectively. In p35 KO Mϕ, only M1 polarized Mϕ showed the ability to form peptide:MHC II complexes, reaching half-maximal IL2 production after 40 minutes. Neither M0 nor M2 p35 KO Mϕ generated many peptide:MHC II complexes, even after 80 minutes (**Figure 6**).

### M1 polarization increases peptide:MHC II stability, which is reduced in absence of p35

**Figure 7:**
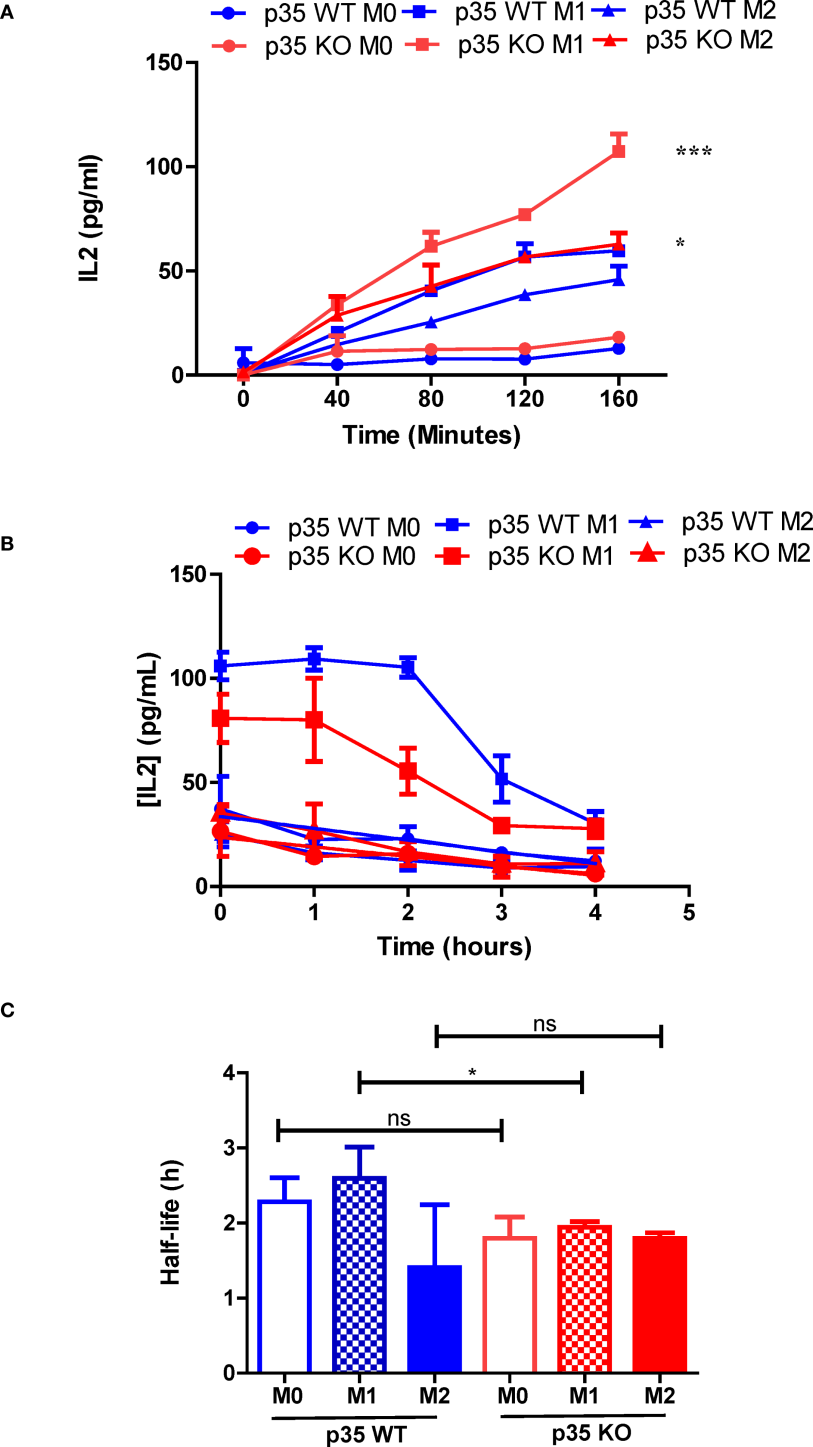
Loss of p35 reduces stability of peptide:MHC II complexes by BMDM. **(A)** BMDM from p35 WT and KO Mϕ were cultured overnight under M0, M1, or M2 polarizing conditions prior to being pulsed with 3 μM OVA_323–339_ for 40, 80, 120, or 160 minutes. BMDM were then washed and fixed, treated with D, L-Lysine prior to addition of DOBW cells. Culture supernatant was collected 24 hours later, frozen and analyzed for IL2 expression by ELISA. **(B)** BMDM from p35 WT and KO Mϕ were cultured overnight under M0, M1, or M2 polarizing conditions prior to being pulsed with 3 μM OVA_323–339_ for 4 hours. BMDM were then washed and fixed 0, 1, 2, 3 or 4 hours thereafter. BMDM were then treated with D, L-Lysine prior to addition of DOBW cells. Culture supernatant was collected 24 hours later, frozen and analyzed for IL2 expression by ELISA. **(C)** Half-life was determined using the formula t_1/2_ = t/log_1/2_(N(t)/N_0_). Comparison between p35 WT and p35 KO were performed using a paired T test. *p < .05; ***p < .001; ns, not significant. Shown is a representative of 4 different experiments.

Our data suggest that the presence of p35 and the Mϕ polarizing conditions could influence the formation of peptide:MHC II complexes. To examine peptide loading, which would reflect surface expression as well as internalization of surface MHC II but not antigen processing, we pulsed WT and KO M0, M1, and M2 Mϕ with 3 μM OVA_323–339_ for varying duration, fixed and then cultured with DOBW for 24 h to evaluate IL2 production by ELISA. Neither M0 populations showed much peptide loading. However, both M1 and M2 Mϕ showed greater peptide loading as reflected by increased surface MHC II expression (**Figures 4A, B**), with KO populations showing significantly higher expression of MHC II as compared to their WT counterparts (**Figure 7**). Next, we surveyed the influence of p35 expression on the stability of peptide:MHC II complexes on the Mϕ surface. We pulsed Mϕ with 10 μM OVA_323–339_ for 4 hours. After washing the cells to remove free peptide, Mϕ were either fixed immediately or fixed at subsequent hourly intervals and then cultured with DOBW for 24 hours to evaluate IL2 production by ELISA. We found that M1 polarized Mϕ showed reduced peptide:MHC II complex stability in p35 KO M1 Mϕ relative to WT Mϕ, while M0 and M2 Mϕ showed minimal stability of peptide:MHC II complex in both p35 KO and WT (**Figures 7C**).

## Discussion

In this study, we investigated the role of CDK5/p35 in Mϕ polarization with results summarized in **Table 1**. First, we evaluated the ability of M-CSF or GM-CSF cultured BMDMs to undergo cytokine-induced polarization. Our investigation showed that, in the presence of LPS alone, GM-BM Mϕ exhibited dramatic upregulation of CD86 compared to M-BM, and this upregulation was partially dependent on the expression of p35. On the other hand, IFNγ alone induced higher CD86 expression in Mϕ cultured under M-BM as compared to GM-BM condition in a p35-independent manner. Combining both LPS and IFNγ resulted in the highest level of CD86 expression in both M-BM and GM-BM Mϕ and, as with IFNγ, this induction was also independent of p35 expression. Thus, LPS-mediated induction of CD86 was seen only in GM-BM with a partial dependence on functional p35.

**Table 1:** Changes due to M1 and M2 polarization in p35 WT and p35 KO BM-derived Mϕ.

Characteristics	p35 WT			p35 KO		
	M0	M1	M2	M0	M1	M2
CD206		–	↑↑↑	–	–	↑↑
ARG-1		–	↑↑↑	–	–	↑
p-STAT3^Ser727^		–	↑↑↑	–	–	↑
OCAR (OXPHOS)		–	–	–	–	↑↑
ECAR (glycosylation)		–	↑	–	↑↑	–
MHC II		↑↑	↑	–	↑↑↑	↑↑
Antigen processing (24 hours)		↑↑	↑			
Bead uptake		↓	↓	↑	↑	↑↑
OVA degradation		–	↑↑	–	–	↑↑
Antigen processing (80 minutes)		↑↑	↑	↓	↑↑	↓
Peptide stability		↑↑	–	–	↑	–

Relative expression compared to WT M0 Mϕ; ↑ or ↓, p<0.05; ↑↑ or ↓↓, p<0.01; ↑↑↑ or ↓↓↓, p<0.001; –, no change.

In tumor cells, CDK5 expression is crucial for IFNγ-induced CD274 expression (46). In the current analysis, however, loss of p35 did not affect CD274 expression in bone marrow-derived Mϕ (BMDM) that were exposed to IFNγ and LPS. CD274 is highly expressed in M1 Mϕ, particularly under inflammatory conditions (47). It functions as a negative regulator of M1 polarization and the production of pro-inflammatory cytokines (48). While previous studies suggested that GM-BM and M-BM correlate with M1 and M2 polarization, our data support the idea that GM-CSF and M-CSF may instead prime Mϕ toward M1 or M2 polarization (49). Additional future investigations are needed to explore these molecular signaling mechanisms that could be mediated by the CDK5/p35 complex.

CD206 expression in M-BM was greatly affected by IL4 but not by p35 expression, suggesting that, at the phenotypic level, p35 was not essential for M2 polarization. There was also no difference in CD206 expression in M-BM versus GM-BM cultured with IL4 (**Supplementary Figure 3**). One major distinction between M1 and M2 Mϕ is the difference in arginine utilization. During M1 polarization iNOS is utilized to convert arginine to L-citrulline and NO that are important for clearing pathogens, while during M2 polarization ARG-1 breaks down arginine to polyamines, L-ornithine, and urea which are important for wound healing (3). ARG-1 expression was decreased during M2 polarization in absence of p35. Regulation of ARG-1 synthesis was shown to be partially dependent on several transcription factors (TF) including STAT3, STAT6, C/EBPβ, PPARγ, PPARδ, IRF8, PU.1, and AP-1 (50). Of these TF, CDK5 has been shown to phosphorylate STAT3 (51) and PPARγ (52). Prior studies implicated the phosphorylation of STAT3 at tyrosine 705 as being important in regulating ARG-1 expression (53), we discovered that loss of p35 reduced phosphorylation at serine 727, suggesting an alternative regulatory pathway of ARG-1 expression involving CDK5/p35. Interestingly, serine 727 phosphorylation of STAT3 is known to cause STAT3 translocation to the mitochondria where it alters metabolism by driving activity of complex I and II of the electron transport chain and enhancing mitochondrial respiration (54). To determine if this decrease in STAT3 phosphorylation seen in p35 KO BMDM can influence mitochondrial metabolism, we evaluated p35 WT and KO Mϕ for ECAR or OCAR under M1 or M2 polarization. We observed the loss of STAT3 serine 727 phosphorylation under M2-culture conditions in p35 KO Mϕ, but the loss of p35 was associated with increased ECAR with M1 Mϕ and increased OCAR with M2 Mϕ (38). Consistent with our phenotypic characterization thus far, we observed a decrease in STAT3 serine 727 phosphorylation among Mϕ in absence of p35.

Our data also suggest that p35 KO Mϕ showed a significant increase in MHC II expression under M0, M1, and M2 conditions (**Figure 4**). To further correlate this increase in MHC II expression with associated Mϕ function, we examined antigen processing by M0, M1, or M2 Mϕ and found that loss of p35 dramatically reduced the ability of all Mϕ populations to process and present Ag after 24 hours, although p35 KO M1 Mϕ did maintain the ability to process antigens at the highest antigen abundance level. Within the p35 WT population, M1 Mϕ showed greatest capacity to process antigen as compared to the M2 Mϕ population. To understand how p35 expression impacted the ability of polarized Mϕ to process antigens, we examined antigen internalization and found that all p35 KO Mϕ populations exhibited greater capability to internalize particulate antigens relative to p35 WT Mϕ, a process that is partially dependent upon high CD206 expression among M2 Mϕ from both p35 WT and p35 KO cultures. We also showed that the degradation of internalized proteins was not affected by p35 expression or Mϕ polarization.

Although activated mitochondria are associated with enhanced antigen processing of peptide:MHC II complexes (55), the ability to degrade antigens does not necessarily reflect the cell’s ability to generate specific peptide fragments that can be complexed with MHC II molecules for subsequent presentation to T cells (56). To test for ability of polarization and p35 expression to influence antigen processing, we assayed Mϕ for its ability to form peptide:MHC II complexes on the cell surface over time. We found M1 Mϕ possessed a significant ability to process and present L-OVA at approximately twice the rate as M0 and M2 in p35 WT Mϕ; M1 Mϕ from p35 KO mice showed ability to rapidly process antigen, reaching a maximum rate after 60 minutes. M0 or M2 Mϕ from p35 KO showed only a modest ability to process and present L-OVA. Furthermore, peptide:MHC II complexes from p35 KO Mϕ had reduced half-life compared to those found on p35 WT Mϕ. Overall, in a 24-hour period p35 WT Mϕ exhibited a prolonged ability to present processed L-OVA to DOBW cells as compared to p35 KO Mϕ. While showing similar rates of degradation, KO Mϕ exhibited an increased ability to internalize particulate Ag, with p35 KO M1 Mϕ showing very good Ag processing ability early on (**Figure 6**). However, this ability by p35 KO M1 Mϕ is not maintained and the subsequent decrease in peptide:MHC II stability (**Figure 7**) resulted in reduced presentation of peptide:MHC II complexes to activate T cells in the long term (**Figure 5**).

Our current study has several limitations. All experiments were conducted using mouse cells *in vitro*, simulating homeostatic conditions. Therefore, our present observation regarding the role of Cdk5/p35 in Mϕ function and polarization requires further investigation *in vivo*, both in animal models and under pathological settings. Additionally, studies using pharmacologic inhibitors of Cdk5/p35 may offer additional valuable insights into the therapeutic potential of modulating this protein complex in Mϕ.

In summary, our current study suggests that disrupting functional CDK5/p35 may inhibit M2 Mϕ from processing and presenting antigens to T cells, potentially resulting in reduced immunosuppressive effects. As tumor-associated Mϕ (TAM) are often characterized as being M2-like, our studies lend scientific rationale in testing a CDK5/p35 inhibitor such as roscovitine or CYC065 in inhibiting TAMs ability to acquire M2-like function while maintaining plasticity towards M1-like phenotype within the TME for the promotion of anti-tumor immunity. Given other documented anti-tumor roles of CDK5 in other immune cell subsets and tumor-associated immune checkpoint molecule expression (22, 23, 46, 57–59), global targeting of CDK5/p35 in tumor-bearing hosts may represent a multi-pronged approach to novel immuno-oncology therapy.

## Data Availability

The raw data supporting the conclusions of this article will be made available by the authors, without undue reservation.

## Supplementary material

Supplementary Figure 1 p35 expression does not influence Mϕ CD86 expression in response to varying doses of LPS or IFNγ. BMDM from p35 WT or p35 KO BM were cultured in the presence of 0, 10, or 100 ng of LPS and IFNγ for 24 hours before being subjected to flow cytometry analysis of CD86 expression. A representative of 4 experiments was shown.

Supplementary Figure 2 p35 is not essential for M1 induction of CD14. **(A)** BMDM from p35 WT or KO BM were cultured under M0, M1, or M2 polarizing conditions for 24 hours before being subjected to flow cytometry analysis of CD14 and TLR4 expression. A representative of 4 experiments was shown, with median fluorescence of CD14 determined. One-Way ANOVA, Turkey multiple comparison. *p <.05; **p <.01; ***p <.001; ns, not significant.

Supplementary Figure 3 Loss of p35 expression does not affect CD206 induction in M2 polarized M-CSF BM-derived Mϕ. **(A)** Both p35 WT and p35 KO were cultured under M2 polarizing conditions and examined for expression of CD206 by flow cytometry. **(B)** The percentage of CD206^+^ cells were enumerated and presented as bar graphs (N = 5), One-Way ANOVA, Turkey multiple comparison: **p <.01; ***p <.001; ns, not significant
