# Supplementary figures and images for: Disruption of CDK5 regulatory subunit 1, p35, limits immunosuppressive M2 macrophages while maintaining functional M1 macrophages
Source: Front Immunol. 2025 Sep 17;16:1584791. doi: 10.3389/fimmu.2025.1584791 (PMC12483927; doi:10.3389/fimmu.2025.1584791)

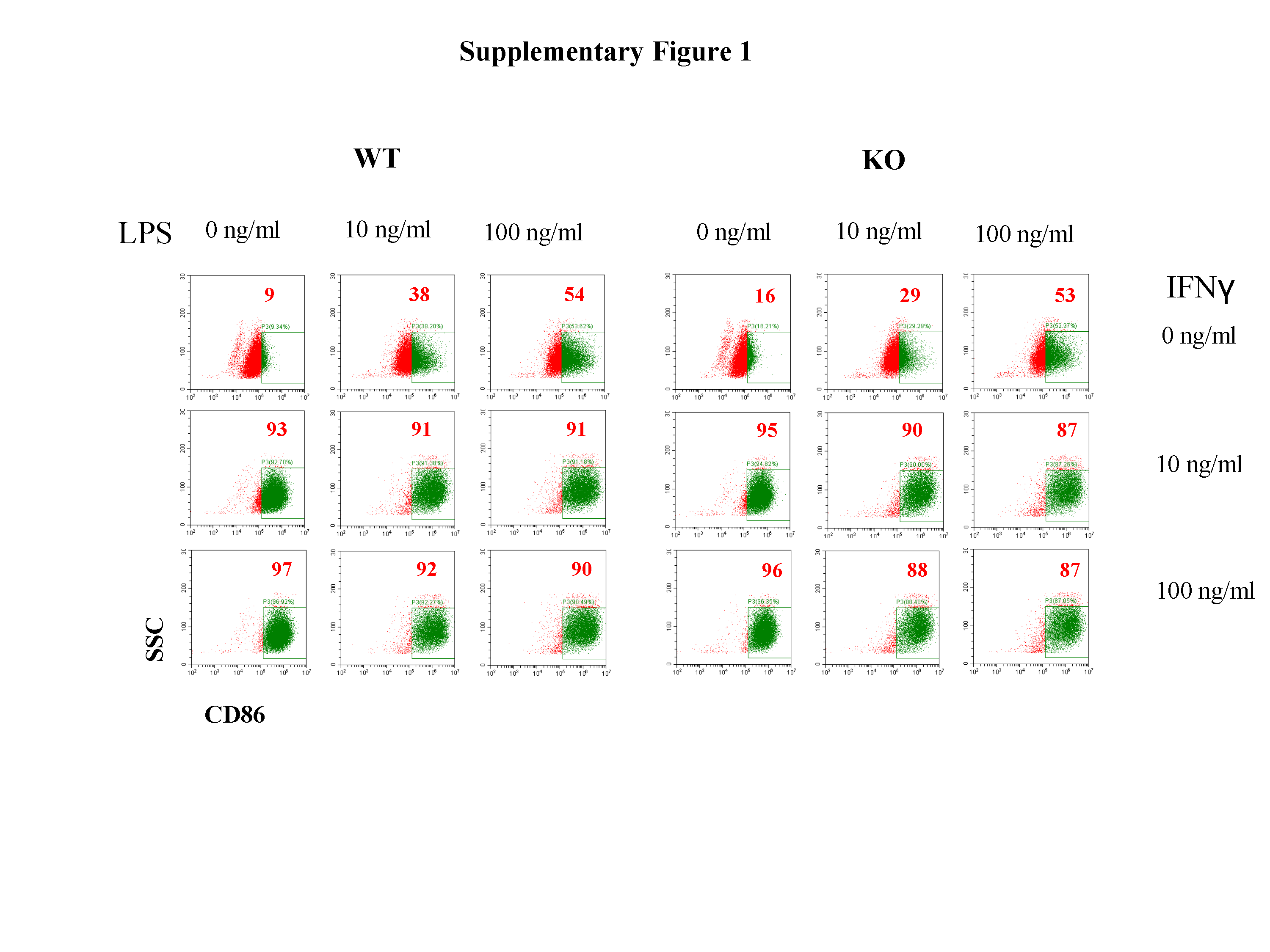

Supplement: Supplementary Figure 1 — p35 expression does not influence Mϕ CD86 expression in response to varying doses of LPS or IFNγ. BMDM from p35 WT or p35 KO BM were cultured in the presence of 0, 10, or 100 ng of LPS and IFNγ for 24 hours before being subjected to flow cytometry analysis of CD86 expression. A representative of 4 experiments was shown. [file Image1.tiff]

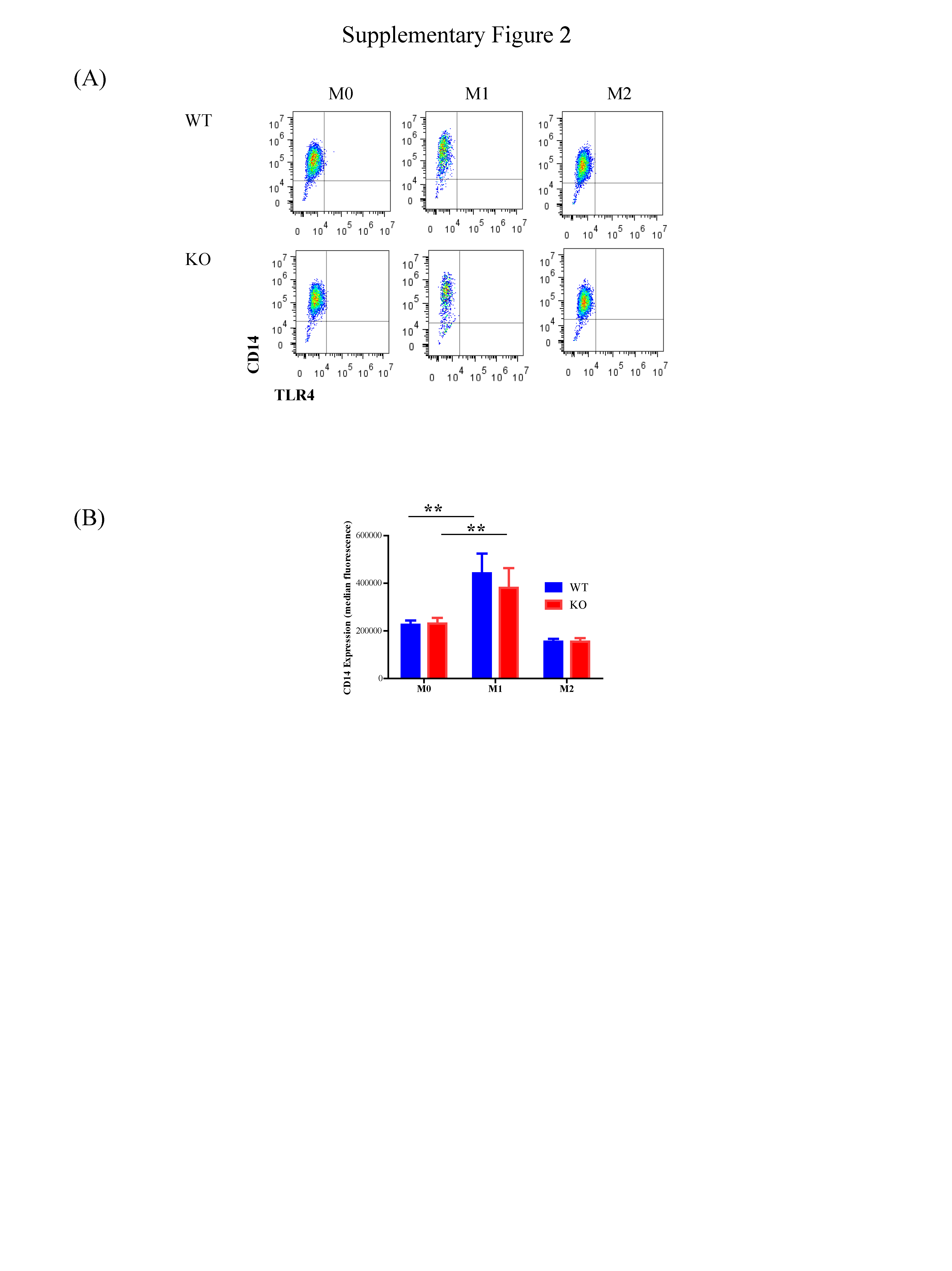

Supplement: Supplementary Figure 2 — p35 is not essential for M1 induction of CD14. (A) BMDM from p35 WT or KO BM were cultured under M0, M1, or M2 polarizing conditions for 24 hours before being subjected to flow cytometry analysis of CD14 and TLR4 expression. A representative of 4 experiments was shown, with median fluorescence of CD14 determined. One-Way ANOVA, Turkey multiple comparison. *p <.05; **p <.01; ***p <.001; ns, not significant. [file Image2.tiff]

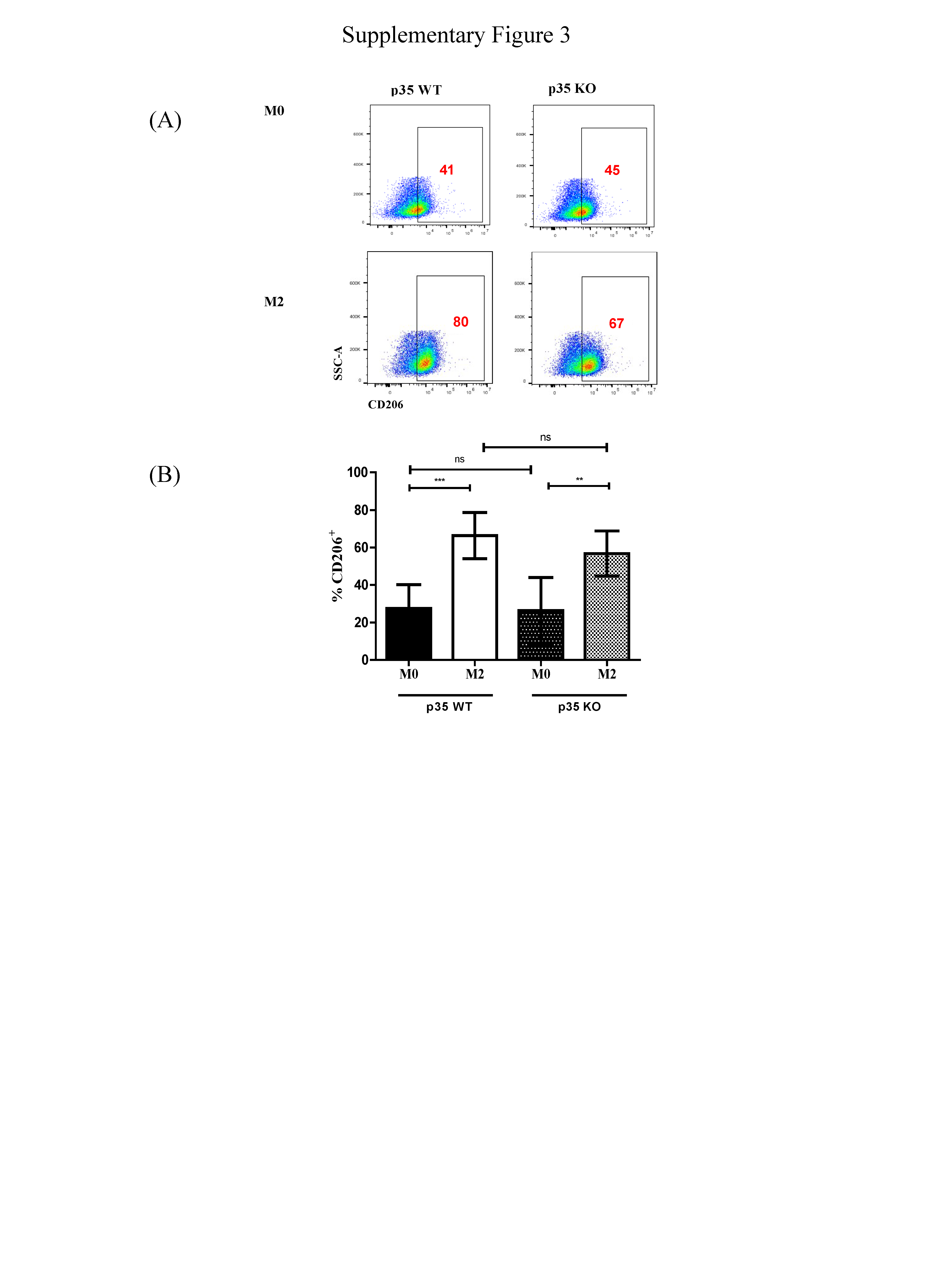

Supplement: Supplementary Figure 3 — Loss of p35 expression does not affect CD206 induction in M2 polarized M-CSF BM-derived Mϕ. (A) Both p35 WT and p35 KO were cultured under M2 polarizing conditions and examined for expression of CD206 by flow cytometry. (B) The percentage of CD206+ cells were enumerated and presented as bar graphs (N = 5), One-Way ANOVA, Turkey multiple comparison: **p <.01; ***p <.001; ns, not significant [file Image3.tiff]
